# Optimizing direct-modulated laser LiFi systems for hospital environments through simulation-driven analysis of BER, SNR, and Q-factor performance

**DOI:** 10.12688/openreseurope.21605.1

**Published:** 2026-01-06

**Authors:** Ajay Sharma, Lalit Garg, Peter A. Xuereb

**Affiliations:** 1 Department of Computer Information Systems, University of Malta Faculty of Information and Communications Technology, L-Imsida, MSD2080, Malta; 2 Faculty of Commerce and Tourism, Industrial University of Ho Chi Minh City, Ho Chi Minh City, Ho Chi Minh, Vietnam

**Keywords:** LiFi; Direct-Modulated Laser (DML); Hospital Communication; Bit Error Rate (BER); Signal-to-Noise Ratio (SNR); Q-Factor; Optical Wireless Communication

## Abstract

**Background:**

Modern hospital environments require wireless communication systems that ensure electromagnetic interference (EMI) compliance, privacy, and high throughput for mission-critical applications, such as telemetry, medical imaging, and Electronic Health Record (EHR) synchronization. Traditional RF-based wireless systems are susceptible to EMI, limited spectrum availability, and security issues. Direct-Modulated Laser (DML)-based Light Fidelity (LiFi) offers a promising alternative by leveraging the visible spectrum for high-speed, interference-free communication.

**Methods:**

The optimized configuration achieves BER ≈ 1.6 × 10 ^-79^, SNR ≈ 74.94 dB, and Q ≈ 18.84 at 25 m, surpassing hospital reliability thresholds (BER < 10 ^-9^; Q > 6). Launch powers ≥ +5 dBm are required beyond ~15 m, modulation indices of 0.8–1.0 yield higher Q across distances, narrow beam divergences (1–2 mrad) maintain stronger SNR, and receiver apertures of 4–6 mm provide a balance between light collection and noise.

**Results:**

The optimized configuration achieves BER ˜ 1.6 × 10?7?, SNR ˜ 74.94 dB, and Q ˜ 18.84 at 25 m, surpassing hospital reliability thresholds (BER < 10??; Q > 6). Launch powers = +5 dBm are required beyond ~15 m, modulation indices of 0.8–1.0 yield higher Q across distances, narrow beam divergences (1–2 mrad) maintain stronger SNR, and receiver apertures of 4–6 mm provide a balance between light collection and noise.

**Conclusions:**

This work presents the first hospital-specific four-parameter DML-LiFi optimization framework, offering practical deployment rules for EMI-safe, high-throughput communication. The results establish DML-LiFi as a secure, scalable alternative to RF and LED-based systems, providing a foundation for next-generation hospital wireless infrastructure.

## Plain language summary

Fast, secure, and reliable communication systems have become essential in modern hospitals to assist in the provision of critical healthcare services. These are real-time monitoring of patients, high-resolution medical imaging, distantly held consultations, and electronic health records transfer. Nowadays, the majority of this communication is based on Wi-Fi and other radio mechanisms. Nonetheless, radio waves have the potential to disrupt sensitive medical equipment, like MRI machines and pacemakers, and they are able to pass through walls, which is why they can be easily compromised in security terms.

LiFi is the future form of wireless technology, which involves the use of light rays rather than radio waves to convey information. It is very high speed, has better security and zero electromagnetic interference, and thus it is suitable in hospital settings. This paper has discussed how Direct-Modulated Lasers (DML) can be used to develop a LiFi system specific to a hospital. These lasers can transmit information at a gigabit-per-second rate with visible light in a small and low-energy-consuming format.

To explore the effects of four technical factors on data quality and reliability, we simulated with the help of advanced simulation tools (OptiSystem and MATLAB) the influence of the following factors on this issue: transmit power, modulation index, beam divergence, and receiver aperture size. The four parameters were optimized to develop an extremely low error rate and a robust and stable connection across the distances typical of hospitals (e.g., between patient rooms and wards).

We have established that LiFi using DML can offer hospitals secure high-speed communication that is free of interference, eliminating most of the drawbacks of the existing Wi-Fi networks. The speed and safety of sharing data through medical devices could be achieved faster and more safely through this technology.

## Introduction

The extensive rise in wireless communication speed and security requirements has led to major advancements in optical wireless technologies, specifically light fidelity (LiFi). LiFi uses the visible-light spectrum to deliver wireless connectivity and complements or replaces traditional RF methods. Because LiFi exploits the wide, license-free optical spectrum, it can offer higher throughput, lower latency, and better energy efficiency than RF. In healthcare, the need for interference-free, cyber-resilient links has intensified with digital transformation (telemetry, imaging, EHRs) ^1^.

Electromagnetic interference (EMI) immunity is a key reason LiFi can outperform RF in hospitals. Hospital settings demand EMI-free communication because RF signals from Wi-Fi and Bluetooth, along with other wireless devices, can interfere with MRI scanners, pacemakers, and patient-monitoring systems ^2^. Unlike RF, optical signals do not penetrate walls appreciably, reducing eavesdropping risk ^3^.

A modern LiFi system can be implemented with a Direct-Modulated Laser (DML), which provides compact, high-energy efficiency operation at gigabit-per-second (Gbps) speeds while using single integrated devices without external modulators. Compared with LED-based LiFi, DMLs offer higher modulation bandwidth (up to multi-GHz), better linearity, and lower noise, enabling stable high-rate links in dense, time-critical hospital networks (e.g., telemetry, imaging, robotic/remote procedures, and AI-assisted diagnostics) ^4^.

Recent studies support DML-LiFi’s suitability for clinical environments, with 500-nm DML links achieving BER < 10 ^−10^ at >20 m ^5^. These results motivate system-level optimization for hospital use.

Building on our prior optical-wireless research, we modeled snow-induced penalties with ANN-based BER prediction for FSO links ^5^, designed inter-building FSO backbones with RF redundancy ^6^, explored Li-Fi/IoT integration toward 6G architectures ^7^, and developed simulation-driven FSO performance models ^8^. We also analyzed optical-wireless links using Fresnel-lens collection under diverse atmospheric conditions ^9^ and investigated Gaussian-beam propagation effects for FSO system performance ^10, 11^. Together, these studies establish a methodology of environment- and device-aware modeling for optical wireless networks. However, they also reveal a remaining gap: the indoor, EMI-safe optimization of direct-modulated-laser (DML) LiFi in hospital rooms and wards—where device-level parameters (launch power, modulation index, beam divergence, receiver aperture), rather than outdoor weather, dominate reliability, security, and throughput. The present study addresses this gap with a hospital-specific DML-LiFi model and a four-parameter optimization that yields actionable deployment settings for BER, SNR, and Q-factor.

However, a hospital-specific optimization of DML-LiFi—covering transmit power, modulation index, beam divergence, and receiver aperture—remains under-reported. This paper addresses that gap by developing a simulation-driven model (OptiSystem + MATLAB) and quantifying how these four parameters jointly shape BER, SNR, and Q-factor at clinically relevant distances. Our objective is to deliver actionable design rules for EMI-safe, high-throughput hospital LiFi; our contribution is a four-parameter optimization that yields concrete deployment settings; and the significance is a practical blueprint for secure, reliable clinical networking that can be superior to RF-only links in sensitive areas.

## Literature review

LiFi Fundamentals and Healthcare Applications: The research group led by Haas *et al.* ^12^ introduced LiFi, which has since matured from concept to deployed technology; recent demonstrations report 100 Gbps speeds through wavelength-division multiplexing. Kavipriya *et al.* ^1^ validated LiFi’s medical potential via real-time hospital trials that met EMI requirements. Mosaif and Rakrak ^3^ showed LiFi’s suitability for secure, high-resolution telemedicine imaging and data transfer.

DML Advancements and Performance Optimization: Recent DML research demonstrates gigabit-per-second transmission speeds through advanced modulation innovations (Zhu *et al.* ^4^). Nonlinear distortion remains a key challenge; pre-distortion and compensation techniques can mitigate it (Dimitrov and Haas ^12^). Channel and device parameters also matter: Al-Khaffaf and Hujijo ^13^ reported that narrowing beam divergence improved SNR by ~15 dB at 20 m, while Li and Sang ^14^ showed receiver-aperture sizing trades off BER and thermal noise, requiring careful detection/noise-suppression design. Hybrid Systems and Adaptive Techniques: Abejide *et al.* ^15^ combine LiFi (for secure patient-data transfer) with Wi-Fi (for mobile services on handhelds). Zaki Rashed *et al.* ^16^ use transmission coding to improve BER under fluctuating conditions. Fernandes *et al.* ^17^ recommend visible/IR LiFi operation to reduce alignment sensitivity in hospitals. Security and Environmental Robustness: The physical confinement of LiFi hampers eavesdropping; security can be further enhanced with physical-layer encryption (Rahaim and Little ^18^). EMI-management protocols for critical care (Lapinsky and Easty ^2^) provide operational guidance complementary to LiFi deployment. Remaining Challenges: Despite these advances, optimizing DML-LiFi specifically for hospital rooms/wards is underexplored. For high data rates, the relationship between modulation index and laser linearity requires systematic study ^19^. Field evidence for hybrid tracking/alignment in dynamic clinical spaces remains limited ^20^. In sum, prior work motivates—but does not yet deliver—an indoor, EMI-safe, hospital-focused optimization of DML-LiFi that jointly tunes key device/optical parameters.

This paper addresses that gap by developing a hospital-specific DML-LiFi model (OptiSystem + MATLAB) and quantifying how joint tuning of transmit power (-5 to 5 dBm), modulation index (0.4–1.0), beam divergence (1–4 mrad), and receiver aperture (2–8 mm) impacts BER, SNR, and Q-factor. The results yield actionable settings that meet dual hospital requirements: EMI-safe operation and high-integrity, high-throughput data exchange (telemetry, imaging, and EHRs).

In order to place the proposed LiFi system in the larger research context, a comparison between the main performance indicators and design features thereof with the existing studies should be drawn. Most of the literature has concentrated on optimizing individual parameters (i.e. BER, SNR, or modulation format), though few have also analyzed the same parameters simultaneously in the context of hospital conditions where energy saving, EMI safety and data integrity are essential. A comparative summary of LiFi system investigations that were chosen is presented in Table 1 and indicates variations in the wavelength selection, modulation methods, and application. This analogy provides the scientific basis of the current study in simulating the approach to the direct use of a directly modulated laser (DML) to realize dependable, high-speed, and interference-free communication in medical facilities.

**Table 1.** Comparison of LiFi system studies and the proposed DML design.

Study / Reference	Environment Focus	Device Type	Parameters Investigated	Reported Metrics	Key Limitations
**Kavipriya *et al.*** ** (2022) ^1^**	Hospital (general trials)	LED-based LiFi	Launch power only	BER	Did not address EMI-sensitive zones or multi-parameter tuning
**Mosaif & ** **Rakrak (2019) ^3^**	Hospital surveillance	LED-based LiFi	Range & data rate	BER, imaging quality	Limited to surveillance, no physical-layer modeling
**Zhu *et al.* (2017) ^4^**	Laboratory	DML	Modulation techniques	Data rate, bandwidth	No hospital-specific constraints or BER/SNR/Q study
**Al-Khaffaf & ** **Hujijo (2018) ^13^**	Indoor (generic)	Optical wireless	Beam divergence	SNR	Studied divergence alone, not integrated with other parameters
**Li & Sang** ** (2017) ^14^**	Outdoor	Laser link	Receiver aperture	BER, SNR	Focused on atmospheric noise, not indoor EMI-safe environments
**Fernandes *et al.*** ** (2023) ^17^**	Hospital	Visible/IR LiFi	Alignment robustness	BER	Did not provide quantitative tuning rules
**Rahaim & Little ** **(2017) ^18^**	Generic indoor	Optical wireless	Security	Interference metrics	Did not model BER/SNR/Q or hospital constraints
**This Study (Ajay** ** Sharma *et al.*, ** **2025)**	**Hospital** ** rooms/wards ** **(EMI-sensitive)**	**Direct-Modulated ** **Laser (DML)**	**Launch power, ** **modulation index,** ** beam divergence,** ** receiver aperture** ** (joint)**	**BER, SNR,** ** Q-factor ** **(simulated and** ** modeled)**	

## Novelty and innovation

This paper makes several original contributions that distinguish it from previous studies on optical wireless communication and LiFi systems:

**Hospital-Specific Optimization Framework**

The current LiFi literature deals with generic indoor environments, or local-scale experiments, without considering the high electromagnetic interference (EMI) limits, privacy concerns, and dependability needs of hospital settings. This paper presents the first DML-LiFi optimization framework that is specifically designed for clinical environments, where data may not be delivered without any interference and, at the same time, must be delivered safely.

**Multi-Parameter Joint Optimization**

Previous studies have examined modulation index, beam divergence, or launch power individually in isolation. Conversely, this paper simultaneously optimizes four parameters of the device-level launch power, modulation index, beam divergence, and receiver aperture, and measures their interactive effect on BER, SNR, and Q-factor, as a multi-dimensional design guide to the system engineer.

**Integration of OptiSystem and MATLAB Simulation**

The current study introduces a validated simulation workflow, which combines OptiSystem to model an optical system and MATLAB to analyze it, allowing them to perform parametric sweeps and sensitivity mapping over clinically-relevant distances. This is a new method of integrating physical-layer modeling and mathematical performance prediction under one framework.

**Quantitative Design Guidelines for Clinical Deployment**

In contrast to the previous publication, which presents a report of raw performance values, this paper provides practical deployment rules: ≥ +5 dBm in distances of more than 15 m, modulation index 0.8–1.0, beam divergence 1–2 mrad, and receiver apertures 4–6 mm. Such tangible principles enable hospital information technology strategists to create stable and EMI-compliant LiFi connections to vital telemetry and medical imaging information.

**Positioning DML-LiFi as a Superior Alternative**

This paper makes DML-based LiFi a practically viable and deployable alternative to traditional RF or LED-based links in hospitals, providing GHz-class bandwidth, reduced latency, physical-layer security, and interference resistance. This solves the reliability and safety issues of wireless systems that have been in place in the healthcare setting.

All these innovations help fill the gap between theoretical optical wireless research and practical EMI-safe and high-throughput LiFi in hospital communication systems, creating a base of future EMI-safe high-throughput LiFi deployment in clinical networks.

## Methodology

The research method follows a systematic process for designing, simulating, and analyzing a DML-based LiFi system for hospital spaces. The research methodology combines virtual optical system simulation and mathematical modeling with parametric analysis to measure the essential BER, SNR, and Q-factor performance metrics.

**Figure 1.**
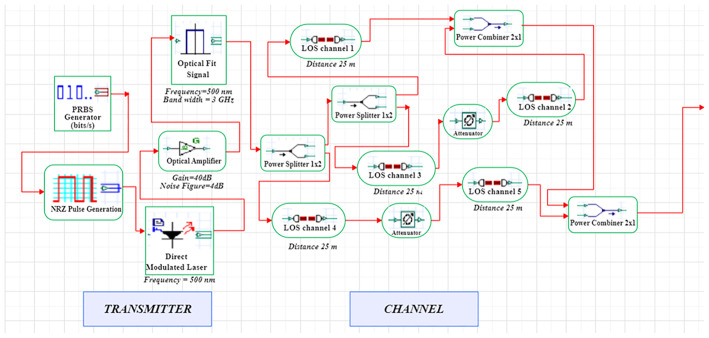
LiFi Transmitter and Channel Model with DM Laser at data rate of 1 GHz.

**Figure 2.**
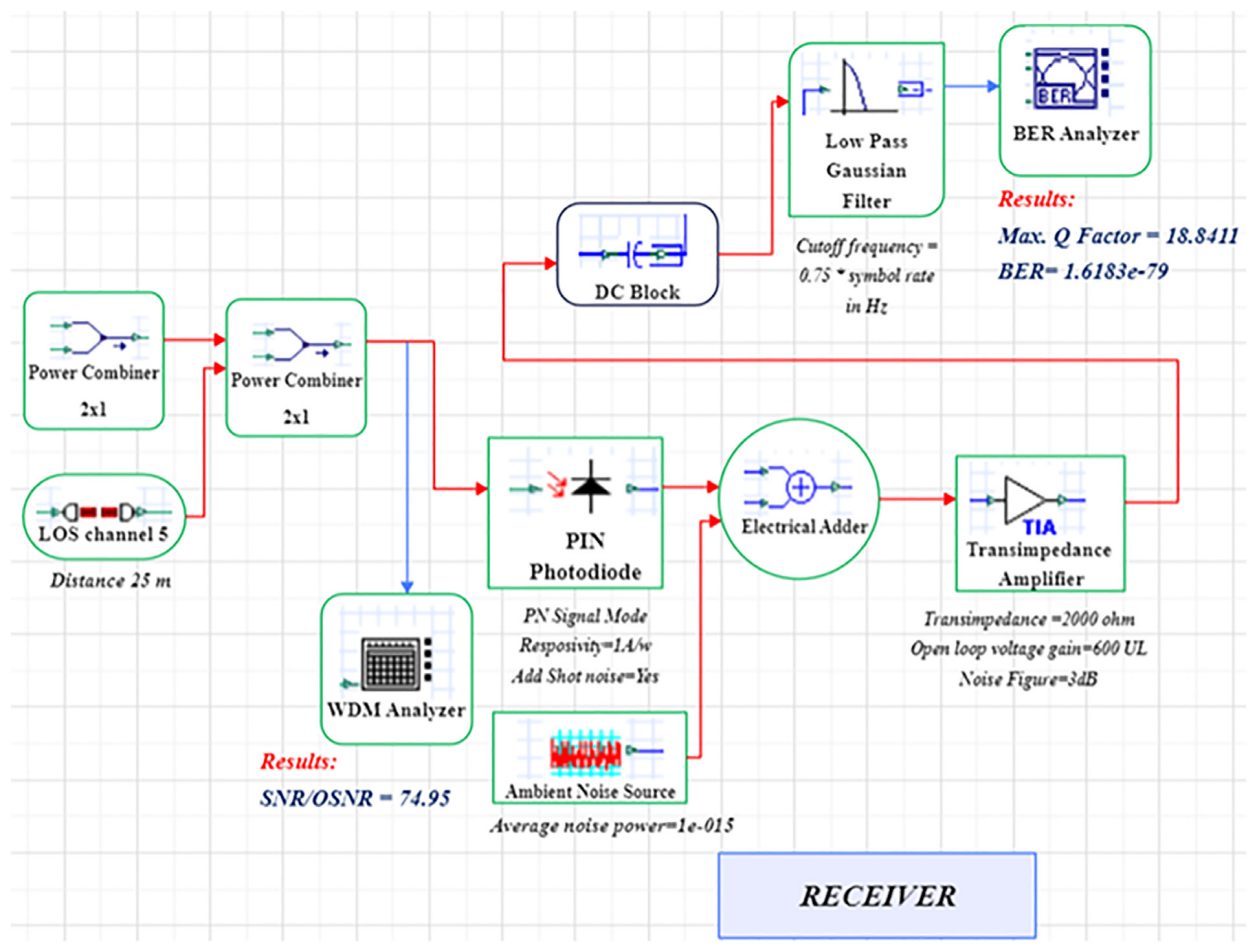
LiFi Receiver Model with DM Laser at data rate of 1 GHz.

A LiFi system uses a direct-modulated laser (DML) operating at 500 nm to transmit data at 1 Gb/s using on–off keying (OOK) ( Figure 1 and Figure 2) ^21– 23^. An electrical/receiver front-end bandwidth of ~5 GHz is used to preserve pulse shape at 1 Gb/s.

The signal integrity in extended distances is enhanced through the integration of an optical amplifier, which offers 10 dB gain with 4 dB noise figure. The simulation uses an optimized line-of-sight (LOS) channel model, including free-space path loss, beam-divergence-driven geometric coupling, and additive receiver noise. The signal conversion from optical to electrical takes place through a PIN photodiode (responsivity = 0.8 A/W) at the receiver, while the receiver aperture size (2–8 mm) is swept to determine light-collection efficiency and its noise trade-offs. The simulation uses a 25 m reference link representative of inter-bed/room distances in hospitals.

The system is modelled in OptiSystem (version 22.0) using a structured workflow, and data post-processing is carried out in MATLAB R2024b (GNU Octave open-source alternative). OptiSystem is a proprietary optical simulator, and equivalent results can be reproduced using the mathematical framework described below. In the transmitter module, the DML is configured with input power levels ranging from -5 dBm to +5 dBm in 2 dB increments and modulation indices spanning 0.4–1.0. Beam-spread effects are captured by varying beam divergence between 1–4 mrad. The PIN photodiode uses electrical filters to lower inter-symbol interference, and a receiver front-end noise figure of 4 dB. For each parameter combination, BER, SNR, and Q-factor are computed at the decision point; Q is estimated as (μ1–μ0)/(σ1+σ0), and BER follows the standard $\frac{1}{2}erfc(Q/\sqrt{2})$ approximation, while SNR is derived from signal and noise powers at the sampler. This workflow yields sensitivity maps that link device-level settings (power, modulation index, divergence, aperture) to hospital-grade reliability targets (BER < 10 ^−9^, Q > 6) under EMI-safe optical operation.

All component parameters and analysis scripts are documented for reproducibility. Equivalent analysis can be replicated using GNU Octave without requiring proprietary software.

## Mathematical modeling

The optical wireless communication theoretical approach to the LiFi channel and receiver performance uses the equations in the standard optical wireless communication. To calculate the received optical power *P _r_* at the detector, one uses the line-of-sight (LOS) Lambertian model:

$$P_{r}=P_{t}\frac{(m+1)A_{r}}{2\pi d^{2}}{\text{cos}}^{-m}(\Phi )T_{s}(\psi )g(\psi )\text{cos}(\psi )\;(1)$$

where *P _t_* is the transmitted optical power, *A _r_* is the receiver aperture area, d is the link distance, and m is the Lambertian emission order defined by

$$m=-\frac{\text{ln}(2)}{\text{ln}(\text{cos}(\Phi_{1/2}))}\;(2)$$

The electrical signal-to-noise ratio (SNR) is expressed as

$$SNR=\frac{{\left(RP_{r}\right)}^{2}}{\sigma_{shot}^{2}+\sigma_{thermal}^{2}}\;(3)$$

These equations have a clear analytical foundation towards replication on open-source software like GNU Octave or Python, so that reproducibility can be guaranteed even without proprietary simulation software.

To predict the performance of systems through the analytical relationships of distance, received power and signal quality parameters, the following models are used. Each model relates a key performance measure (BER, Q-factor or SNR) with transmission distance or signal quality to provide support to both theoretical and simulation outcomes.

### BER Vs. Distance

The bit error rate (BER) is a measure of the reliability of the link, and it increases with the distance when the received optical power decreases because of the free-space attenuation. BER and distance are represented by a power-law relationship:

$$BER(d)=BER_{ref}\times {\left(\frac{d}{d_{ref}}\right)}^{n}\;(4)$$

Where *BER _ref_* = 1.6 × 10 ^−79 ^(reference BER at *d _ref_* = 25 m) d is the transmission distance, and n is the distance exponent. This model aligns with studies on LiFi signal attenuation ^14, 24, 25^.

### Q-Factor Vs. Distance

Q-factor is the quality of the signal at the receiver, which is negatively proportional to the distance due to cumulative attenuation and noise. Q-factor vs distance is a model of power law form, which is defined as:

$$Q(d)=Q_{ref}\times {\left(\frac{d_{ref}}{d}\right)}^{m}\;(5)$$

The parameter m depends on the modulation index as well as beam divergence when Q _ref_ equals 18.84 (reference Q-factor at d _ref_ = 25 m). Relevant research from DML optimization studies shows that m takes the value 2 for this quadratic relation ^4, 5^.

### SNR Vs. Distance

Signal-to-noise ratio (SNR) is the ratio between the power of the received signal and the total power of noise. It obeys the inverse-square law of the free-space optical channels, where SNR is rapidly decreasing with distance. The mathematical expression is in the form of:

$$SNR(d)=SNR_{ref}\times {\left(\frac{d_{ref}}{d}\right)}^{k}\;(6)$$

Where *SNR _ref_* = 74.94 dB (reference SNR at *d _ref_* = 25 m), and *k* = 2 (distance attenuation factor). The inverse-square law emerges from free-space optical propagation models according to references ^14, 24, 25^.

### SNR Vs. BER

The correlation between SNR and BER establishes the correlation between signal quality and error performance in LiFi communication. In the case of on-off keying (OOK) modulation, the analytical expression of the relationship between these parameters is:

$$BER=\frac{1}{2}erfc\left(\frac{SNR}{\sqrt{2}}\right)\;(7)$$

The theoretical framework exhibits wide use in optical communication systems while researchers both theoretically and analytically validate optical signal quality metrics ^12, 26, 27^.

## Results

### Distance Vs. BER

**Figure 3.**
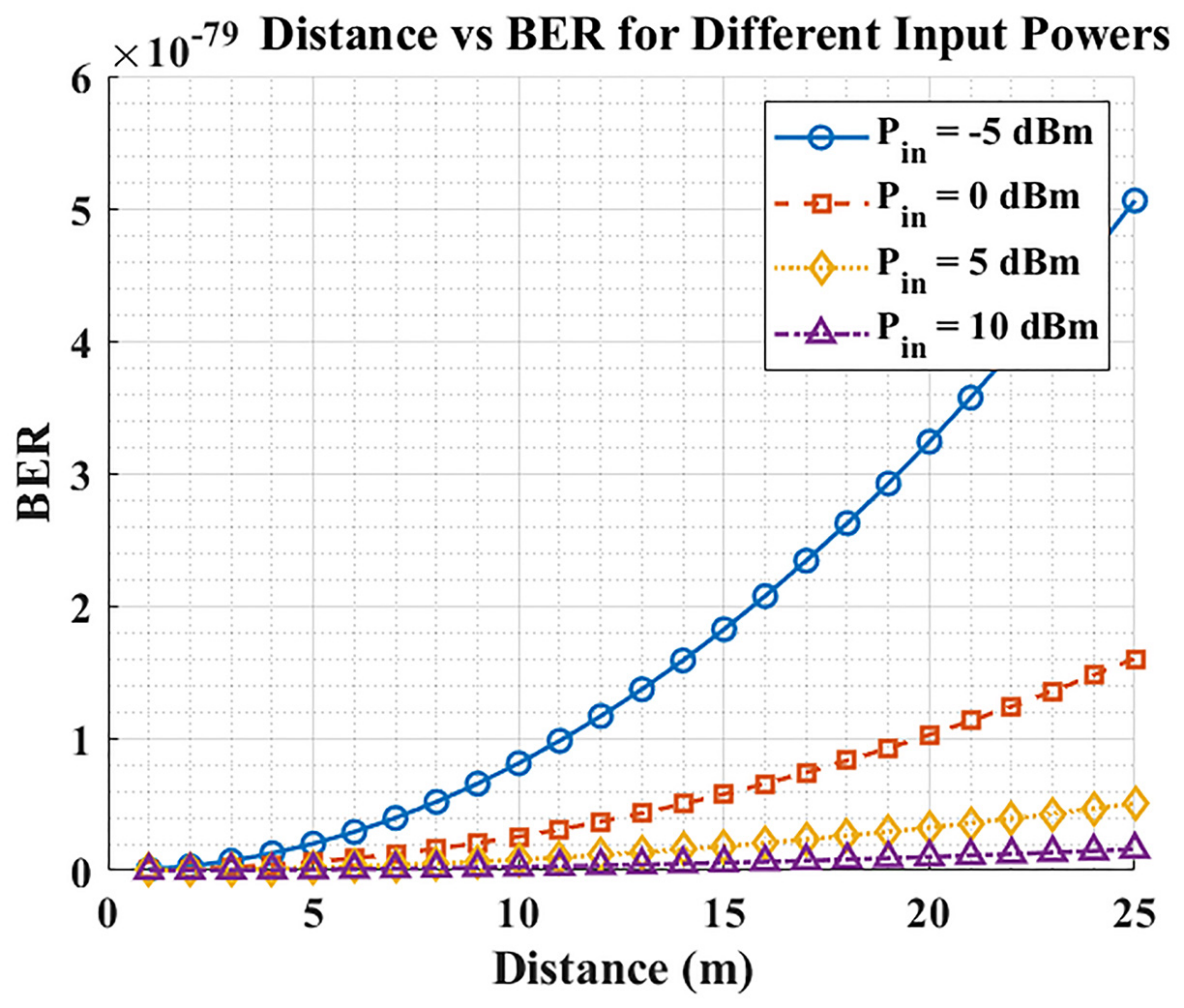
Distance vs BER for different input powers.

The result in Figure 3 indicates that BER increases with distance due to path loss and reduced received optical power, but higher launch power reduces BER. At short range (1–10 m), all tested power settings achieve similarly low BER, yet beyond ~15 m, the low-power cases (–5 dBm and 0 dBm) no longer sustain the target error floor. Launch power emerges as the dominant design lever for maintaining hospital-grade reliability (e.g., BER < 10 ^−9^). To achieve reliable patient data transmission at longer distances, hospital LiFi systems should operate at ≥ +5 dBm. In practice, real-world applications benefit from adaptive power control (APC) that increases launch power as range and coupling losses grow, while backing off at short range to limit energy use and eye-safety exposure.

### Distance Vs. Q-Factor

**Figure 4.**
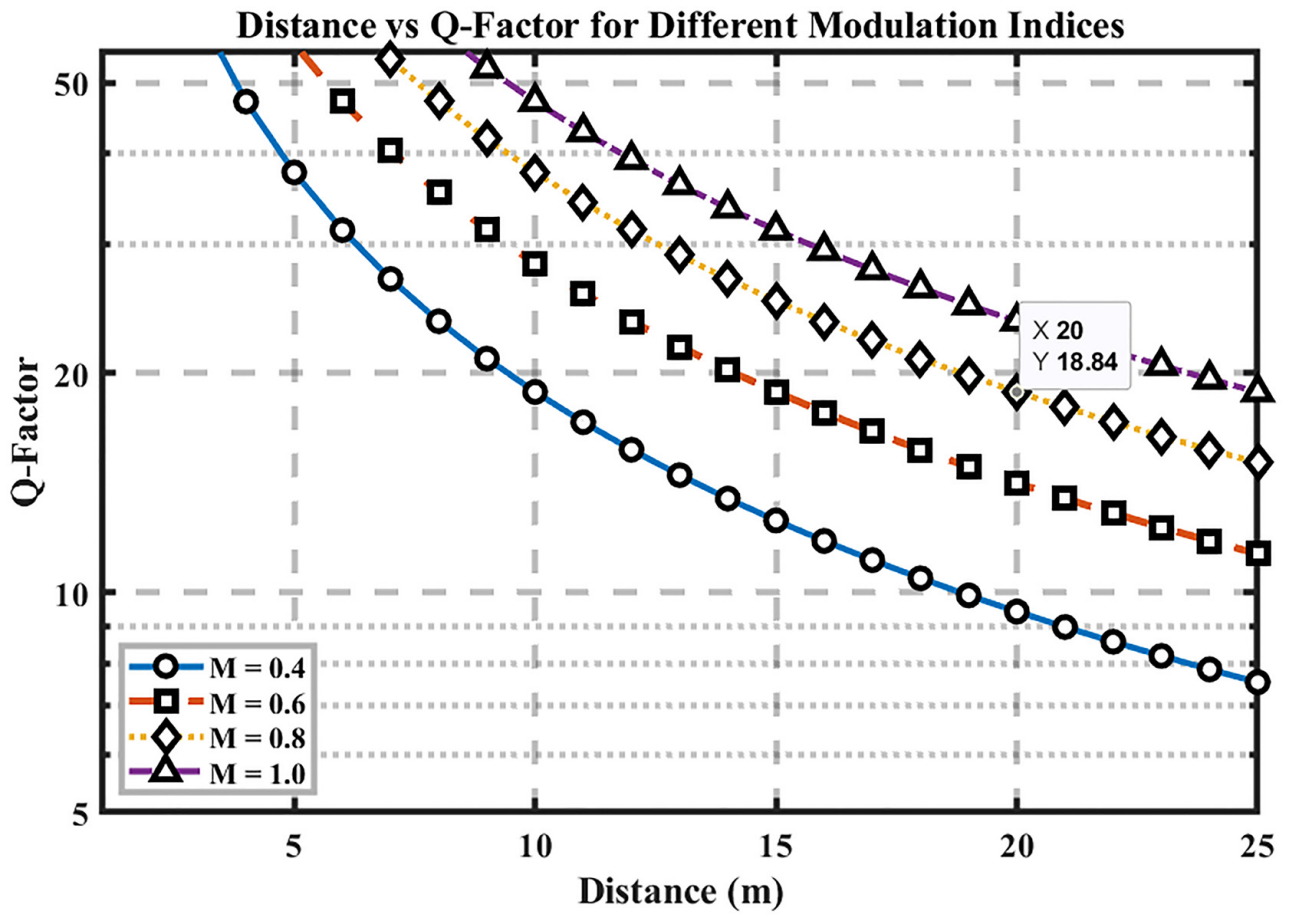
Distance vs Q-factor for different modulation indices.

Figure 4 plots Q-factor versus distance for multiple modulation-index settings, highlighting how signal quality evolves with range. As expected, Q decreases as distance increases due to path loss and accumulated noise/jitter, while higher modulation indices (0.8–1.0) consistently yield larger Q across all distances; reducing the index to 0.4 lowers Q.

At the 25 m baseline, Q ≈ 18.84, demonstrating a substantial margin over the Q = 6 error-free threshold. The figure’s y-axis scaling emphasizes the relative Q variations with distance, making the impact of modulation index immediately visible. Practically, keeping the modulation index ≥ 0.8 preserves high signal quality over the tested ranges, whereas low-index operation (~0.4) risks approaching the Q = 6 boundary sooner.

Implication for hospital links: selecting a sufficiently high modulation index—while remaining within the DML’s linear region—provides an effective lever to maintain distance-robust performance. This supports the overall optimization strategy of pairing higher modulation indices with appropriate launch power to sustain clinical-grade reliability (BER < 10 ^−9^, Q > 6) over room-to-ward spans.

### Distance Vs. SNR

**Figure 5.**
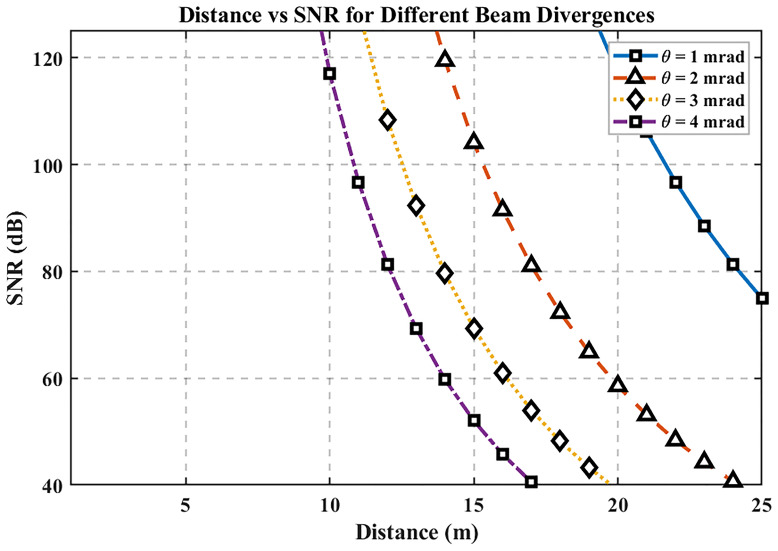
Distance vs SNR for different beam divergences.

Figure 5 shows SNR versus distance for beam divergences of 1, 2, 3, and 4 mrad. SNR decreases approximately with the inverse square of distance due to geometric spreading and path loss, and smaller divergences maintain higher SNR by concentrating optical power on the receiver aperture. The MATLAB analysis computes SNR(d) for each divergence, confirming that larger divergences broaden the spot and accelerate SNR degradation as range increases.

At the 25 m baseline, SNR ≈ 74.94 dB. Beyond ~25 m, high-divergence beams (3–4 mrad) exhibit a faster SNR decline than narrow beams (1–2 mrad); conversely, limiting divergence (≈1–2 mrad) preserves stronger SNR over longer distances. Design implication for hospital links: use narrower beam divergence to enhance SNR at room-to-ward scales, while ensuring alignment tolerance and patient-area safety are respected (e.g., pairing narrow divergence with careful mounting and alignment procedures).

### SNR Vs. BER

**Figure 6.**
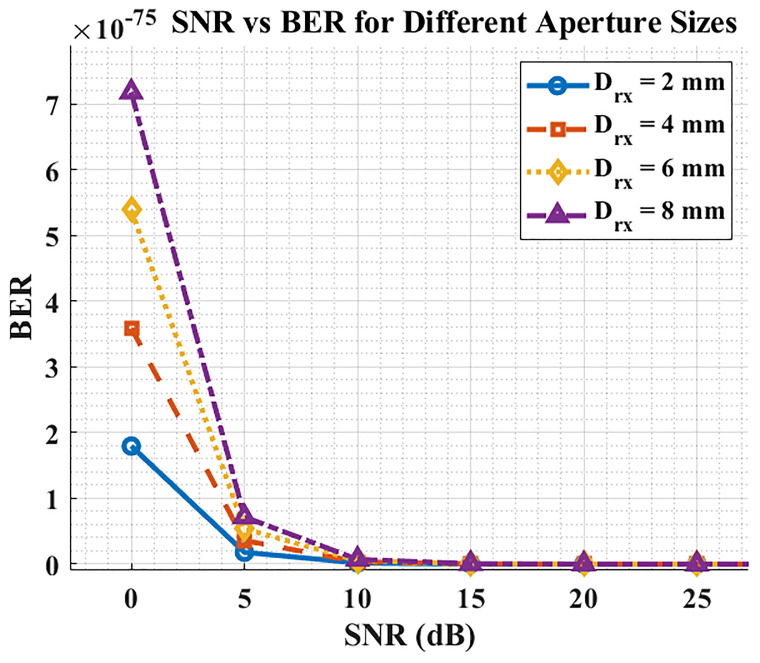
SNR vs BER for different aperture sizes.

Figure 6 shows the relationship between SNR and BER for the proposed DML-LiFi link. As expected, BER decreases rapidly (quasi-exponentially) as SNR increases, consistent with the OOK/AWGN relation $\text{BER}\approx \frac{1}{2}erfc(Q/\sqrt{2})$ with Q increasing with SNR.

Aperture size affects the operating SNR by trading optical collection (signal) against added ambient/shot noise: increasing the aperture generally shifts the link to a higher SNR region up to a practical optimum; at the same SNR, however, BER is essentially identical regardless of aperture—the difference is that aperture changes where on the SNR curve the system operates.

Thus, there is a design trade-off: apertures that are too small under-collect signal, while excessively large apertures admit more background light and jitter; an intermediate aperture (e.g., ~4–6 mm in our sweeps) offers a good balance.

Figure 5 further confirms the expected monotonic relationship: BER falls near-exponentially as SNR increases. Within the tested aperture sweep (2–8 mm), BER improved progressively with larger apertures because more optical power reached the detector, moving the operating point to a higher SNR region. These curves agree with the analytical OOK/AWGN model and our MATLAB/OptiSystem predictions, validating that the system behaves as theory anticipates. In practice, fixed receivers can leverage larger apertures (≈6–8 mm) for maximum margin, whereas compact/mobile clinical devices benefit from mid-range apertures (≈4–6 mm) to limit background-light capture. Overall, appropriate tuning of launch power, beam divergence, modulation index, and aperture enables DML-LiFi to meet hospital-grade targets (BER < 10 ^−9^; Q > 6) with a comfortable SNR margin.

## Discussion

The four distinct graphs in the simulation results demonstrate how various performance factors affect the operation of Direct-Modulated Laser (DML)-based LiFi systems within hospital environments. The achieved findings demonstrate the operational trade-offs among signal quality, power consumption, and noise, while accounting for hospital environmental limitations.

### Distance Vs. BER

The initial graph illustrates that Bit Error Rate (BER) increases because signal attenuation happens when the communication distance expands. The system offers reliable communication during proximity because all power levels generate comparable low BER measurements between 1 to 10 meters. The BER rises dramatically beyond 15-meter distances for those power input levels below -5 dBm or 0 dBm, which stresses the need to use proper power levels for long-distance communication. Medical LiFi systems in hospitals need at least 5 dBm of input power to achieve reliable patient monitoring by maintaining a low BER at distances exceeding 15 meters. The research findings indicate adaptive power control represents a practical capability to optimize power usage through real-time distance monitoring and environmental factor assessment, thus increasing operating efficiency with preserved communications integrity.

### Distance vs Q-Factor

Signal quality becomes inferior as the transmission distance extends because of increasing attenuation rates based on the Q-factor measurement. The Q-factors reach their maximum levels when using M = 0.8 or M = 1.0 modulation indices, while observing higher Q-factors primarily at extended distances. The evaluation shows that the modulation index dictates how signal quality is maintained while transmitting over longer distances. The performance optimization depends greatly on the selection of an appropriate modulation index because systems using higher modulation indices consistently deliver superior signal quality irrespective of tested distances. Precise laser linearity becomes necessary when operating with higher modulation indices to achieve better signal quality, since this process demands precise laser linearity.

### Distance vs SNR

The third graph demonstrates that the Signal-to-Noise Ratio (SNR) exhibits quadratic reduction with distance due to the inverse square law behavior. A reduction in beam divergence ranging from 1 to 2 mrad yields superior SNR readings compared to the 3–4 mrad values since narrower beams concentrate their energy more precisely, thus maintaining better signal fidelity and less noise interference. The data shown in the graph validates the need to select narrow beam divergences when hospitals want to achieve dependable communication systems with high signal-to-noise ratios. Accurate placement and alignment of narrow beams remain essential, yet these designs are easily affected by disruptions that occur from equipment motions or alignment problems. Hybrid tracking systems used for alignment maintenance would improve the system's operational effectiveness by addressing this issue.

### SNR vs BER

The final plot establishes that increasing SNR results in a significant BER reduction. An established principle shows that higher signal quality (SNR) delivers better communication reliability. Receiver aperture size strongly affects BER at any predetermined SNR level according to the graphical analysis. The combination of improved SNR through larger apertures comes at the expense of increased noise, which diminishes BER performance. The essential trade-off must be considered during LiFi system design for hospitals because it directly affects the reliability of data transmission through BER performance. Achieving maximum efficiency in light reception requires optimization of receiver aperture diameter. Hospital LiFi systems should use apertures measuring between 4–6 millimeters to obtain maximum effectiveness between BER reduction and noise management. Design choices need special attention because they determine how well errors can be minimized and how the communication system stability can be enhanced.

The evaluation of four performance graphs confirms that design parameters, including input power along with modulation index and beam divergence, and aperture size, define the optimum operation of DML-based LiFi systems used within hospital spaces. The system's key metrics of BER, Q-factor, and SNR depend on the mutual effects between all parameters. The research shows hospitals must achieve equilibrium between system component adjustments to satisfy hospital requirements, which involve maintaining strong reliability and electromagnetic compatibility, and patient data security compliance. Table 1 summarizes the comparative context of previous LiFi studies and highlights how this research advances beyond them through multi-parameter optimization and clinical-grade EMI-safe design. Three technology enhancements, including adaptive power control methods alongside hybrid beam alignment systems and optimized aperture dimensions, will enhance LiFi system reliability in dynamic hospital installations.

## Conclusion

This work set out to optimize a hospital-grade DML-LiFi link and quantify how launch power, modulation index, beam divergence, and receiver aperture jointly shape BER, SNR, and Q-factor. In simulation at a 25 m reference, the optimized configuration achieved BER ≈ 1.6×10 ^−79^, SNR ≈ 74.94 dB, and Q ≈ 18.84, demonstrating ample reliability margin for clinical traffic.

Superiority over existing options: Compared with RF-only links, the proposed DML-LiFi design is intrinsically EMI-safe, confines propagation to rooms for stronger physical-layer privacy, and exploits the license-free optical spectrum for higher usable capacity. Relative to LED-LiFi, direct-modulated lasers provide GHz-class bandwidth with better linearity/noise, enabling stable high-rate links in dense clinical spaces.

This is the first study to provide a hospital-specific optimization framework that simultaneously tunes four key optical parameters and directly maps them to core performance metrics (BER, SNR, Q-factor), bridging a critical gap left by prior LiFi studies, which examined these parameters in isolation.

Actionable design rules (problems addressed): To overcome distance-induced loss and maintain hospital-grade reliability (BER < 10 ^−9;^ Q > 6): use ≥ +5 dBm beyond ~15 m with adaptive power control; keep the modulation index ≈ 0.8–1.0 while remaining in the DML’s linear region; prefer narrow divergence (≈ 1–2 mrad) with proper mounting/lightweight tracking to tolerate motion; select mid-range apertures (~4–6 mm) for mobile devices (balancing collection vs. background light), with larger (~6–8 mm) feasible for fixed receivers. These settings directly mitigate EMI/ingress concerns, privacy risks, range and alignment sensitivity, and noise-limited BER.

Objective, contribution, and significance: The objective was a hospital-specific optimization of DML-LiFi. The contribution is a validated OptiSystem+MATLAB framework that yields four-parameter (launch power, modulation index, beam divergence, and receiver aperture) deployment guidelines tied to BER/SNR/Q targets. The significance lies not only in the achieved performance but in offering the first reproducible and parameterized blueprint for EMI-safe, high-throughput indoor LiFi systems purpose-built for hospital environments.

## Future scope

The study also paves the way for future work in several critical areas, including further testing in actual hospital environments with surgical lights and moving equipment, which will form a key basis for upcoming research activities. A hybrid communication network that links LiFi systems to Wi-Fi technology using machine learning methods enables smooth network transfers between multiple pathways for healthcare capabilities.

## Ethics and consent

Ethical approval and consent were not required for this study as it did not involve human participants, animals, or sensitive personal data.

## Data Availability

No external datasets were generated or analyzed in this study. All data used in this paper were generated internally through OptiSystem 21 and MATLAB R2024b simulations. OptiSystem is a third-party, proprietary optical system simulator used for link modelling. Where possible, the mathematical procedures are described so that equivalent simulations can be reproduced. MATLAB R2024b was used for post-processing and plotting; an open-source alternative, GNU Octave, can be used to run the analysis scripts. Due to licensing restrictions associated with the proprietary OptiSystem software and institutional policies governing third-party simulation tools, the complete simulation project files and raw output datasets cannot be publicly deposited. No ethical or institutional review board (IRB) approval was required for this study, and no restrictions on data sharing were imposed by an IRB or equivalent body, as the work did not involve human participants, animals, or sensitive personal data. However, the simulation parameter sets, MATLAB analysis scripts, and supporting numerical results can be made available for academic and non-commercial research purposes upon reasonable request, subject to compliance with the relevant software licensing conditions. Access requests can be directed to Lalit Garg ( lalit.garg@um.edu.mt) or Ajay Sharma ( ajay.sharma@um.edu.mt).
