# Supplementary material for: Optimizing direct-modulated laser LiFi systems for hospital environments through simulation-driven analysis of BER, SNR, and Q-factor performance
Source: Open Res Eur. 2026 Apr 19;6:13. Originally published 2026 Jan 6. [Version 3] doi: 10.12688/openreseurope.21605.3 (PMC13126009; doi:10.12688/openreseurope.21605.3)
Supplement: Peer review history

# Reviewer report: Reviewer response for version 3

**DOI:** 10.21956/openreseurope.25447.r72770

**Published:** 2026-04-30

**Authors:** Urvashi Chaudhary

**Affiliations:** 1 COPELABS, Universidade Lusófona, Lisboa, Portugal

This report provides an evaluation of the research article titled " **Optimizing direct-modulated laser LiFi systems for hospital environments through simulation-driven analysis of BER, SNR, and Q-factor performance**".

**Article Summary**

 The study proposes a four-parameter optimization framework for Direct-Modulated Laser (DML)-based LiFi systems specifically tailored for hospital settings. Using OptiSystem and MATLAB, the authors simulated the joint effects of launch power, modulation index, beam divergence, and receiver aperture on performance metrics like Bit Error Rate (BER), Signal-to-Noise Ratio (SNR), and Q-factor. The research identifies theoretical "upper-bound" performance trends, suggesting that DML-LiFi can meet high reliability standards (BER < 10^{-9}) under idealized, well-aligned line-of-sight conditions in EMI-sensitive environments.

**Presented accurately and cites current literature?**

**Partly**

 The literature review was enhanced in Version 3 to include hybrid LiFi/RF systems and FSO-based recovery. However, reviewers noted initial gaps in comparing it to LED-based or VCSEL systems.

**Study design appropriate and academic merit?**

**Yes**

 The study design is considered appropriate for a theoretical/simulation-based analysis. It offers merit by exploring multi-parameter joint effects rather than isolated variables.

**Sufficient methodology for replication?**

**Partly**

 While the mathematical procedures and simulation workflow (OptiSystem/MATLAB) are described, proprietary licensing prevents the sharing of raw project files.

**Statistical analysis appropriate?**

**Yes**

 The analysis follows established OOK/AWGN models, and the simulation results are internally consistent with analytical trends.

**Source data available for reproducibility?**

**Partly**

 No external datasets were used; data was generated internally. Full project files are not public due to institutional policies and proprietary software restrictions.

**Conclusions supported by results?**

**Partly**

 Earlier versions overclaimed reliability (e.g., BER of 10^{-79}). While Version 3 moderates these to "theoretical feasibility," they remain based on "best-case" scenarios.

Is the study design appropriate and does the work have academic merit?

Yes

Is the work clearly and accurately presented and does it cite the current literature?

Yes

If applicable, is the statistical analysis and its interpretation appropriate?

Yes

Are all the source data underlying the results available to ensure full reproducibility?

Yes

Are the conclusions drawn adequately supported by the results?

Yes

Are sufficient details of methods and analysis provided to allow replication by others?

Yes

Reviewer Expertise:

Wireless Communication

I confirm that I have read this submission and believe that I have an appropriate level of expertise to confirm that it is of an acceptable scientific standard.

# Reviewer report: Reviewer response for version 3

**DOI:** 10.21956/openreseurope.25447.r72653

**Published:** 2026-04-28

**Authors:** Jean-Paul Linnartz

**Affiliations:** 1 Eindhoven University of Technology, Eindhoven, The Netherlands

I thank the authors for taking my previous comments carefully into account. The paper is much more mature now.

Is the study design appropriate and does the work have academic merit?

Partly

Is the work clearly and accurately presented and does it cite the current literature?

Partly

If applicable, is the statistical analysis and its interpretation appropriate?

Not applicable

Are all the source data underlying the results available to ensure full reproducibility?

Partly

Are the conclusions drawn adequately supported by the results?

No

Are sufficient details of methods and analysis provided to allow replication by others?

Partly

Reviewer Expertise:

Wireless Communication

I confirm that I have read this submission and believe that I have an appropriate level of expertise to confirm that it is of an acceptable scientific standard.

# Reviewer report: Reviewer response for version 3

**DOI:** 10.21956/openreseurope.25447.r72654

**Published:** 2026-04-23

**Authors:** Chun Sing Lai

**Affiliations:** 1 Tianjin University, Tianjin, Tianjin, China

I have no further comment.

Is the study design appropriate and does the work have academic merit?

Yes

Is the work clearly and accurately presented and does it cite the current literature?

Partly

If applicable, is the statistical analysis and its interpretation appropriate?

Yes

Are all the source data underlying the results available to ensure full reproducibility?

Partly

Are the conclusions drawn adequately supported by the results?

Partly

Are sufficient details of methods and analysis provided to allow replication by others?

Partly

Reviewer Expertise:

Devices and Circuits, Smart Cities, Energy Systems

I confirm that I have read this submission and believe that I have an appropriate level of expertise to confirm that it is of an acceptable scientific standard.

# Reviewer report: Reviewer response for version 2

**DOI:** 10.21956/openreseurope.25041.r70529

**Published:** 2026-03-05

**Authors:** Chun Sing Lai

**Affiliations:** 1 Tianjin University, Tianjin, Tianjin, China

The authors claimed that “The four parameters were optimized to develop an extremely low error rate and a robust and stable connection across the distances typical of hospitals (e.g., between

 patient rooms and wards).” For this highly nonlinear and multi-objective problem, please explain how the proposed technique can achieve the optimal solution, i.e., how do the authors know that the soultion is optimal? Which optimization algorithm is used?

 Figures 1 and 2 present the transmitter and receiver models, however, it is not clear what is the state of the art and how it is innovative as compared to previous models. The authors may make a comparison figure or add clear annotations on where the innovation is.

 For a multidimensional problem, i.e., BER, SNR, and Q-factor, it may be more appropriate to plot 3-dimensional plots or overlapped figures of the plots to identify the optimal configuration of the system, by identifying how do the four parameters affect the system performance.

 There is a lack of comparison with the state of the art methods and different device type. Some quantitative comparisons would add value to the work.

 Minor comments:

 The symbols for equations should be properly formatted, please check the italic fonts for consistency.

 The figures must be properly made to ensure results are presented clearly. For example, Fig. 5’s legend hides some results. Fig. 4 the databox hides the line.

Is the study design appropriate and does the work have academic merit?

Yes

Is the work clearly and accurately presented and does it cite the current literature?

Partly

If applicable, is the statistical analysis and its interpretation appropriate?

Yes

Are all the source data underlying the results available to ensure full reproducibility?

Partly

Are the conclusions drawn adequately supported by the results?

Partly

Are sufficient details of methods and analysis provided to allow replication by others?

Partly

Reviewer Expertise:

Devices and Circuits, Smart Cities, Energy Systems

I confirm that I have read this submission and believe that I have an appropriate level of expertise to state that I do not consider it to be of an acceptable scientific standard, for reasons outlined above.

# Response

**Published:** 2026-03-25

**Authors:** Ajay Sharma

**Affiliations:** Computer Information Systems, University of Malta Faculty of Information and Communications Technology, Msida, Malta

We are grateful to the reviewer for the elaborate and constructive feedback. The feedback has helped us enhance the clarity, rigour, and placement of the manuscript. Point-by-point, we respond below.

**Reviewer Comment** *“The authors claimed that “The four parameters were optimized to develop an extremely low error rate and a robust and stable connection across the distances typical of hospitals (e.g., between patient rooms and wards).” For this highly nonlinear and multi-objective problem, please explain how the proposed technique can achieve the optimal solution, i.e., how do the authors know that the soultion is optimal? Which optimization algorithm is used.”*

**Author Response:** In the modified document, we explain that the term optimized refers to a systematic parametric search and sensitivity analysis over predefined intervals of launch power, modulation index, beam divergence, and receiver aperture. No mathematical nonlinear or multi-objective optimization (e.g., genetic algorithm, particle swarm optimization, convex optimization) algorithm was used. The configuration that performs best within the explored parametric space can be defined as optimal and is the parameter set that achieves the highest level of analytical reliability (e.g., BER < 10 ^-9^ and Q > 6) and has robust SNR margins under idealised detector-noise-limited assumptions. We now make it quite clear that this does not imply a mathematically global optimum. Instead, it is an analytically preferable upper-bound design in the explored design space. The paper has also been edited to prevent overstatement.

**Reviewer Comment** *“Figures 1 and 2 present the transmitter and receiver models, however, it is not clear what is the state of the art and how it is innovative as compared to previous models. The authors may make a comparison figure or add clear annotations on where the innovation is.”*

**Author Response:** The updated document now makes it clear that the transmitter and receiver hardware blocks are not inherently new architectures. This work has been innovative in terms of the structured joint four-parameter sensitivity framework that has been specifically designed to suit hospital sized indoor environment. In contrast to other literature that examine individual parameters independently, this paper will combine joint tuning of launch power, modulation index, beam divergence, and receiver aperture and map the combined effect on BER, SNR, and Q-factor with hospital-based limitations. Table 1 has been reinforced to make a more distinct comparison with the previous studies, showing the difference in parameter range, type of devices used, and no hospital specific multi-parameter analysis in previous research.

**Reviewer Comment** *“For a multidimensional problem, i.e., BER, SNR, and Q-factor, it may be more appropriate to plot 3-dimensional plots or overlapped figures of the plots to identify the optimal configuration of the system, by identifying how do the four parameters affect the system performance.”*

**Author Response:** This is a helpful recommendation by the reviewer. The present research uses the structured two-dimensional sensitivity plots to isolate and interpret the influencing effect of the specific parameters when the other parameters are under controlled assumptions. We concur that multi-objective visualization of Pareto-fronts and three-dimensional response surfaces would be more useful in understanding trade-offs. We have included in the revised manuscript a statement in the Future Scope section that will say that future work will consider:

- Three-dimensional response-surface analysis,
- Multi-objective optimization by Pareto-fronts,
- Strict evolutionary optimization algorithms.

The current study forms a first-order analysis foundation, which can be expanded in the future in multidimensional optimization research.

**Reviewer Comment** *“There is a lack of comparison with the state of the art methods and different device type. Some quantitative comparisons would add value to the work.”*

**Author Response** In the updated manuscript, quantitative and qualitative comparison has been made clearer and is now included:

- LED-based LiFi systems (wider coverage but lower modulation bandwidth),
- Direct-modulated lasers (higher bandwidth but narrower coverage),
- VCSEL array approaches (alignment mitigation but increased complexity),
- Hybrid LiFi–RF systems for resilience.

We have made clear that the suggested DML-based frame does not have any implied universal superiority but rather offers a parameterized analytical baseline in idealized conditions of the indoor of a hospital.

**Reviewer Comment** *“The symbols for equations should be properly formatted. Please check the italic fonts for consistency.”*

**Author Response** All equations have been reformatted with consistent italicized variables and corrected fraction structures.

**Reviewer Comment** *“The figures must be properly made to ensure results are presented clearly. For example, Fig. 5’s legend hides some results. Fig. 4 the databox hides the line.”*

**Author Response** All figures have been revised to improve clarity.

**Evaluation Summary**

**Questions** **Comment** *Is the work clearly and accurately presented and does it cite the current literature?*

*Partly*

**Response:** To make sure correct presentation, the literature review has been enlarged, comparative positioning has been made clear, and claims have been moderated.

**Comment** *Are sufficient details of methods and analysis provided to allow replication by others?*

*Partly*

**Response:** The paper has now explained the methodology of the parametric sweep, assumptions of analysis, the scope of the noise model and conditions of reproducibility. In the optimization procedure, the details are provided in a manner that enables one to independently reproduce the analytical trends in MATLAB.

**Comment** *Are all the source data underlying the results available to ensure full reproducibility?*

*Partly*

**Response:** We made it clear that proprietary OptiSystem project files may not be publicly deposited as a result of licensing restrictions, but that parameter sets, mathematical formulae, and MATLAB scripts can be made available on reasonable academic request.

**Comment** *Are the conclusions drawn adequately supported by the results?*

*Partly*

**Response:** All claims have been moderated. The conclusions have now been made clearer in the Discussion section to report that they represent upper-bound analytical performance in idealized conditions and not the reliability of deployment. The aspects of practical implementation are already recognized as future work.

**Closing Statement** The reviewer has written very constructive and technically insightful remarks, for which we thank him. The paper has undergone significant revisions to help explain optimization methodology, enhance comparative positioning, enhance the quality of figures, and tone down claims. In our opinion, the revisions enhance the clarity of the work and its academic rigor in a major way.

# Reviewer report: Reviewer response for version 1

**DOI:** 10.21956/openreseurope.23373.r68125

**Published:** 2026-02-06

**Authors:** Jean-Paul Linnartz

**Affiliations:** 1 Eindhoven University of Technology, Eindhoven, The Netherlands

Direct modulation of a laser can be an attractive approach to allow a cost down and miniaturization of optical wireless systems. That makes the work interesting and relevant.

 However in the present form, the paper overclaims to an unacceptable degree. Most importantly, the scientific community in LiFi must not advise health care providers to adopt LiFi in hospitals, solely based on the findings in this report. The paper seems to suggest that the results are a breakthrough and that the reliability of optical links is flawless (10^-79). This strong claim is not sufficiently substantiated.

 It seems like an overclaim to conclude “”this paper makes DML-based LiFi a practically viable”” if the study is (only) a simplified theoretical model. The idea of DML is interesting and the theoretical analysis has merit, but the overall claim that authors made a breakthrough in making LiFi in hospitals feasible is unsufficiently supported. In any revision, claims should be limited to something like

  "in theory, in a well-aligned line-for-sight link, the link budget and SNR for a directly modulated laser of a few mWatt seems more than sufficient to support OOK at 1 Gbit/s over 25 meter if the beam divergence is below a millirad".

 The authors must realize that claiming a BER of 10^-79 may be interpreted by many readers (including myself) a sign of a lack of a maturity in reliability engineering: A BER of 10^-79 suggests that not a single bit error would have occurred in any instance of billions of such systems in parallel that all started transmitting since to beginning of the universe. Engineers educated in reliability of systems understand that many more aspects need to be considered to achieve a typical 5 nines (99,999 %) reliability of a link. Relying on an excessive SNR is not adequate.

 Deep gaussian tails (certainly Q>18) are notoriously inaccurate as an error model. Extrapolating a BER versus SNR curve based on Gaussian assumptions (!?) to claim exorbitant reliability sounds a bit naïve. I am afraid that such numbers put the trustworthiness of the paper into question, already once reading the abstract. (at least that was my feeling after reading just the abstract).

 Surprisingly, the section after: “”Building on our prior optical-wireless research,   ..“” reveals that the authors have a much more mature perspective on these various aspects than shown in the current paper.

 Statements such as:

 “”A modern LiFi system can be implemented with a Direct-Modulated Laser (DML), which provides compact, high-energy efficiency operation at gigabit-per-second (Gbps) speeds while using single integrated devices without external modulators.”” needs a justification. These do not follow from results in this paper. References are needed about power efficiency (relative to radio, to LEDs, ?). If the SNR (Q) is so excessive, then much lower power would also suffice!? Secondly, the low power relies on covering a just single client device in small area of a few tens of cm's.

  I am not convinced that there are no EMI challenges in LiFi: photodetectors are notoriously sensitive in picking up RF signals, and modulating currents into low impedance lasers often leads to unwanted emissions in the radio spectrum. I am not too convinced with a paper claiming absence of EMI without studying these effects explicitly  and only relying on the fact that the intended (!) emissions are not in the radio spectrum. Receiver noise caused by picking up RF signals (man-made noise, non-Gaussian) is usually orders of magnitude larger than additive noise in the amplifier. Even reaching 10^-12 is hard in a practical setting, for EMI reasons.

 Shot noise highly depends on the environmental light. This effect is not quantified except for mentioning some precautions of optical shielding. 500 nm is in the visible range of hospital illumination, thus environmental light causes significant shot noise. 

 The assumed noise figure of 4 dB needs justification when used as a transimpedance amplifier for an optical system. the use of this as a noise model (borrowing assumptions of 50 Ohm terminations in RF signal amplification?) requires further elaboration for TIA connected to photo diodes. 

 I do not understand how in (4) the BER can be a function of distance to the power n, rather than via the erfc(distance ^ n) . How are the error function (erfc) expressions used, if eq. (4) seems to model BER in a different manner. 

 Eq 4 seems a misuse of Turbulence-limited FSO channels and not appropriate for detector-noise limited indoor channels. ISI is not modelled, and certain at low BER these effects become a limiting factor.

 Is the noise white as assumed in the models? Typical detectors have a low-pass response. Correcting this response in a TIA usually results in enhanced noise power densities that increase with frequency. (the only gentle roll off above 1 GHz seems to accept a lot of noise enhancements) In particular, the use of a Lambertian sensitivity model for the photo diode detector implies the use of relatively large (naked) dye area (no lenses), thus high capacitances. It is unclear what the aspect of angle of arrival is brought into the model, as this aspect of misalignment is not described.

 “”current study introduces a validated simulation workflow””. But how is it validated?

 Regarding: “”This solves the reliability and safety issues of wireless systems that have been in place in the healthcare setting.”” 

 1) I do not read how the authors resolved the blockage of a line of sight, which is the most frequently heard objection against LiFi.  

 2) I do not read about whether or not moving / portable devices need to supported in hospital settings. Would doctors or nurses want to calibrate the directivity of a laser while preparing to a medical intervention?  

 3)  I do not read how the authors resolved eye-safety issues with lasers at 500 nm, this in the visible range.

 Compared to studies with LEDs, similar bit rates have been achieved but with much wider coverage (10 m2) than the laser spot (1 mrad at 25 m illuminates only a small spot). LED give comparable bit/s per watt/m^2 and wide coverage seems more appropriate for the application.

 The references seem a bit biased towards FSO and lack links to paper on providing reliable LiFi coverage indoors, including MIMO to resolve l.o.s. blockage issues, including solutions based on (ITU or IEEE) standards  for which interfacing electronics ICs are available.

 There is also quite some literature on using arrays of VSCELS. These can also be seen as directly modulated lasers, and bring a solution to the issue of aligning the beam direction. I am not convinced that the work advances the state of art if benchmarked against published work on VCSEL arrays.

 Because of these considerations I cannot recommend exposure of the paper in its current form to the readers.

Is the study design appropriate and does the work have academic merit?

Partly

Is the work clearly and accurately presented and does it cite the current literature?

Partly

If applicable, is the statistical analysis and its interpretation appropriate?

Not applicable

Are all the source data underlying the results available to ensure full reproducibility?

Partly

Are the conclusions drawn adequately supported by the results?

No

Are sufficient details of methods and analysis provided to allow replication by others?

Partly

Reviewer Expertise:

I have published many papers in the field of LiFi

I confirm that I have read this submission and believe that I have an appropriate level of expertise to state that I do not consider it to be of an acceptable scientific standard, for reasons outlined above.

# Response

**Published:** 2026-02-09

**Authors:** Ajay Sharma

**Affiliations:** Computer Information Systems, University of Malta Faculty of Information and Communications Technology, Msida, Malta

**Response to Reviewer 2 Comments**

 We are grateful to the reviewer, who has examined, in detail and rigorously and constructively, the subject. We are grateful to the reviewer that he has read so carefully, and we admit that some of the assertions in the original manuscript are exaggerated. We have, in turn, significantly amended the manuscript in order to capture scope, redress exaggeration, and make all conclusions within the framework of the theoretical and simulation-based nature of the study. Each comment is reproduced below and explained how it has been met.

**Reviewer Comment** *“Direct modulation of a laser can be an attractive approach to allow a cost down and miniaturization of optical wireless systems. That makes the work interesting and relevant. However, in the present form, the paper overclaims to an unacceptable degree. Most importantly, the scientific community in LiFi must not advise health care providers to adopt LiFi in hospitals solely based on the findings in this report.”*

**Author Response:** We fully agree. The manuscript has been updated all over (Abstract, Introduction, Novelty, Results, Discussion, and Conclusion) to clearly indicate that the manuscript offers upper-bound performance analysis based on theory and simulations only. Anything that hints at the adoption of a hospital or deployment preparedness has been eliminated.

**Reviewer Comment** *“The paper seems to suggest that the results are a breakthrough and that the reliability of optical links is flawless (10^-79). This strong claim is not sufficiently substantiated.”*

**Author Response:** This is an issue that is completely dealt with. The Abstract, Results, Discussion, Mathematical Modeling and Conclusion have been cleared of all signs of BER ≈ 10 ^-9^. BER is now always defined as well below the analytical level (e.g., BER < 10 ^-9^), and it is clearly identified as an analytical lower limit based on Gaussian-noise modelling, and not operational reliability.

**Reviewer Comment** *“It seems like an overclaim to conclude ‘this paper makes DML-based LiFi a practically viable’ if the study is (only) a simplified theoretical model.”*

**Author Response:** We agree. All expressions that purported to be viable in practice, deployable, or a solution to hospital reliability have been eliminated or weakened. The rewritten text makes many attempts to explain that the paper only judges on the theoretical feasibility in idealized conditions of line-of-sight and detector-noise-limited.

**Reviewer Comment** *“In any revision, claims should be limited to something like ‘in theory, in a well-aligned line-for-sight link…’”*

**Author Response** This very framing has been utilized across the edited text. The Abstract, Methodology, Results, Discussion, and Conclusion now clearly express themselves using the terminology of in theory, under idealized assumptions, and upper-bound analytical performance.

**Reviewer Comment** *“The authors must realize that claiming a BER of 10^-79 may be interpreted … as a sign of a lack of maturity in reliability engineering.”*

**Author Response** We do consider this fact and completely agree with it. The updated manuscript acknowledges clearly the shortcomings of Gaussian tail extrapolation at large Q-factors, and the values of BER are no longer regarded as reliability measures, but rather as analytical ones. Claims of reliability engineering are eliminated.

**Reviewer Comment** *“Deep gaussian tails (certainly Q>18) are notoriously inaccurate as an error model.”*

**Author Response** This weakness is now clearly mentioned in the Methodology section as well as in the Mathematical Modeling section. It is explicitly stated that the erfc-based formulation of the BER is an approximation that becomes less valid at large Q, and the results are put in context.

**Reviewer Comment** *“Statements such as ‘A modern LiFi system can be implemented with a Direct-Modulated Laser (DML)…’ needs a justification.”*

**Author Response** This is an updated sentence. There are no longer careless claims of energy efficiency, EMI immunity, and better than RF or LEDs, and literature has been included to support the claims. This study could not make justification to some extent, and hence this claim was eliminated.

**Reviewer Comment** *“I am not convinced that there are no EMI challenges in LiFi…”*

**Author Response** We agree. Any alleged claims of the operation being free of EMI have now changed to “EMI-safe in terms of intended optical emissions”. The updated version takes explicit responsibility for EMI pickup in photodetectors and front-end electronics as a significant pragmatic constraint that is not represented in this work.

**Reviewer Comment** *“Shot noise highly depends on the environmental light… 500 nm is in the visible range…”*

**Author Response** This is a limitation that is now clearly stated. It is indicated clearly that the noise model is detector-noise-limited, and the effect of ambient-light-induced shot noise is not in-scope, and needs to be studied in the future.

**Reviewer Comment** *“The assumed noise figure of 4 dB needs justification…”*

**Author Response** The noise figure is no longer used as a realistic TIA model, but rather as a simplified or idealistic assumption. The manuscript explains that the frequency-dependent noise and the practical TIA behaviour are not modelled.

**Reviewer Comment** *“I do not understand how in (4) the BER can be a function of distance to the power n…”*

**Author Response** It has been reinterpreted as equation (4) and is clearly stated as an empirical trend model, rather than a law of physical BER. This has been represented by a clear separation in the manuscript between the expressions of erfc-based BER and distance-dependent trend fitting that are only utilized to aid in the interpretation of the results of the simulations.

**Reviewer Comment** *“ISI is not modelled…”*

**Author Response** This has now clearly mentioned in the Methodology and Mathematical Modeling sections.

**Reviewer Comment** *“‘current study introduces a validated simulation workflow’. But how is it validated?”*

**Author Response** The term “validated” has been replaced with “reproducible” throughout the manuscript. Validation is now defined strictly as internal consistency between analytical trends and simulation outputs, not experimental verification.

**Reviewer Comment** *“I do not read how the authors resolved the blockage of a line of sight… moving / portable devices… eye-safety issues…”*

**Author Response** We concur that they are essential matters. The updated Discussion and Conclusion clearly indicate that line-of-sight blockage, mobility, and eye safety are not resolved in this study and need to be addressed in hybrid architectures and experimental studies in the future.

**Reviewer Comment** *“Compared to studies with LEDs… VCSEL arrays…”*

**Author Response** Literature Review and Discussion have been extended to note LED-based LiFi, MIMO LiFi, and VCSEL array designs, and explain that DML-LiFi is a single design point of many, each with trade-offs.

**Reviewer Comment** *“Because of these considerations, I cannot recommend exposure of the paper in its current form to the readers.”*

**Author Response:** We appreciate such an honest evaluation by the reviewer. The manuscript has been revised on a large scale to accommodate all issues of concern. Exaggerated assertions have been eliminated, scope has been defined, constraints have been clearly stated, and conclusions are now fully substantiated by the theoretical analysis provided.

**Reviewer Summary Questions**

- *Is the work clearly and accurately presented, and does it cite the current literature?*
**Revised to address gaps and balance citations.**
- *Is the study design appropriate, and does the work have academic merit?*
**Clarified as a theoretical and simulation-based study.**
- *Are sufficient details provided for replication?*
**Clarified as numerical reproducibility, not experimental validation.**
- *Are the conclusions supported by the results?*
**Rewritten to match analytical scope.**

We sincerely thank the reviewer for highlighting critical issues related to reliability engineering, modeling assumptions, and scientific responsibility. The revised manuscript reflects a more mature, careful, and accurate presentation of the work, and we believe it is now suitable for consideration as a theoretical and simulation-based contribution to LiFi research.

# Response

**Published:** 2026-02-23

**Authors:** Ajay Sharma

**Affiliations:** Computer Information Systems, University of Malta Faculty of Information and Communications Technology, Msida, Malta

We are grateful to the reviewer, who has examined, in detail and rigorously, and constructively, the subject. We are grateful to the reviewer that he has read so carefully, and we admit that some of the assertions in the original manuscript are exaggerated. We have, in turn, significantly amended the manuscript in order to capture scope, redress exaggeration, and make all conclusions within the framework of the theoretical and simulation-based nature of the study. Each comment is reproduced below and explained how it has been met. **Reviewer Comment** *“Direct modulation of a laser can be an attractive approach to allow a cost down and miniaturization of optical wireless systems. That makes the work interesting and relevant. However, in the present form, the paper overclaims to an unacceptable degree. Most importantly, the scientific community in LiFi must not advise health care providers to adopt LiFi in hospitals, solely based on the findings in this report.*

*”* **Author Response:** We fully agree. The manuscript has been updated all over (Abstract, Introduction, Novelty, Results, Discussion, and Conclusion) to clearly indicate that the manuscript offers upper-bound performance analysis based on theory and simulations only. Anything that hints at the adoption of a hospital or deployment preparedness has been eliminated.

**Reviewer Comment** *“The paper seems to suggest that the results are a breakthrough and that the reliability of optical links is flawless (10^-79). This strong claim is not sufficiently substantiated.”*

**Author Response:** This is an issue that is completely dealt with. The Abstract, Results, Discussion, Mathematical Modeling and Conclusion have been cleared of all signs of BER ≈ 10 ^-9^. BER is now always defined as well below the analytical level (e.g., BER < 10 ^-9^), and it is clearly identified as an analytical lower limit based on Gaussian-noise modelling, and not operational reliability.

**Reviewer Comment** *“It seems like an overclaim to conclude ‘this paper makes DML-based LiFi a practically viable’ if the study is (only) a simplified theoretical model.”*

**Author Response:** We agree. All expressions that purported to be viable in practice, deployable, or a solution to hospital reliability have been eliminated or weakened. The rewritten text makes many attempts to explain that the paper only judges on the theoretical feasibility in idealized conditions of line-of-sight and detector-noise-limited.

**Reviewer Comment** *“In any revision, claims should be limited to something like ‘in theory, in a well-aligned line-for-sight link…’”*

**Author Response** This very framing has been utilized across the edited text. The Abstract, Methodology, Results, Discussion and Conclusion now clearly express themselves using terminology of in theory, under idealized assumptions, and upper-bound analytical performance.

**Reviewer Comment** *“The authors must realize that claiming a BER of 10^-79 may be interpreted … as a sign of a lack of maturity in reliability engineering.”*

**Author Response** We do consider this fact and completely agree with it. The updated manuscript acknowledges clearly the shortcomings of Gaussian tail extrapolation at large Q-factors, and the values of BER are no longer regarded as reliability measures, but rather as analytical ones. Claims of reliability engineering are eliminated.

**Reviewer Comment** *“Deep gaussian tails (certainly Q>18) are notoriously inaccurate as an error model.”*

**Author Response** This weakness is now clearly mentioned in the Methodology section as well as in the Mathematical Modeling section. It is explicitly stated that the erfc-based formulation of the BER is an approximation that becomes less valid at large Q, and the results are put in context.

**Reviewer Comment** *“Statements such as ‘A modern LiFi system can be implemented with a Direct-Modulated Laser (DML)…’ needs a justification.”*

**Author Response** This is an updated sentence. There are no longer careless claims of energy efficiency, EMI immunity, and better than RF or LEDs, and literature has been included to support the claims. This study could not make justification to some extent, and hence this claim was eliminated.

**Reviewer Comment** *“I am not convinced that there are no EMI challenges in LiFi…”*

**Author Response** We agree. Any alleged claims of the operation being free of EMI have now changed to “EMI-safe in terms of intended optical emissions”. The updated version takes explicit responsibility for EMI pickup in photodetectors and front-end electronics as a significant pragmatic constraint that is not represented in this work.

**Reviewer Comment** *“Shot noise highly depends on the environmental light… 500 nm is in the visible range…”*

**Author Response** This is a limitation that is now clearly stated. It is indicated clearly that the noise model is detector-noise-limited, and the effect of ambient-light-induced shot noise is not in-scope, and needs to be studied in the future.

**Reviewer Comment** *“The assumed noise figure of 4 dB needs justification…”*

**Author Response** The noise figure is no longer used as a realistic TIA model, but rather as a simplified or idealistic assumption. The manuscript explains that the frequency-dependent noise and the practical TIA behaviour are not modelled.

**Reviewer Comment** *“I do not understand how in (4) the BER can be a function of distance to the power n…”*

**Author Response** It has been reinterpreted as equation (4) and is clearly stated as an empirical trend model, rather than a law of physical BER. This has been represented by a clear separation in the manuscript between the expressions of erfc-based BER and distance-dependent trend fitting that are only utilized to aid in the interpretation of the results of the simulations.

**Reviewer Comment** *“ISI is not modelled…”*

**Author Response** This has now clearly mentioned in the Methodology and Mathematical Modeling sections.

**Reviewer Comment** *“‘current study introduces a validated simulation workflow’. But how is it validated?”*

**Author Response** The term “validated” has been replaced with “reproducible” throughout the manuscript. Validation is now defined strictly as internal consistency between analytical trends and simulation outputs, not experimental verification.

**Reviewer Comment** *“I do not read how the authors resolved the blockage of a line of sight… moving / portable devices… eye-safety issues…”*

**Author Response** We concur that they are essential matters. The updated Discussion and Conclusion clearly indicate that line-of-sight blockage, mobility, and eye safety are not resolved in this study and need to be addressed in hybrid architectures and experimental studies in the future.

**Reviewer Comment** *“Compared to studies with LEDs… VCSEL arrays…”*

**Author Response** Literature Review and Discussion have been extended to note LED-based LiFi, MIMO LiFi, and VCSEL array designs, and explain that DML-LiFi is a single design point of many, each with trade-offs.

**Reviewer Comment** *“Because of these considerations, I cannot recommend exposure of the paper in its current form to the readers.”*

**Author Response:** We appreciate such an honest evaluation by the reviewer. The manuscript has been revised on a large scale to accommodate all issues of concern. Exaggerated assertions have been eliminated, scope has been defined, constraints have been clearly stated, and conclusions are now fully substantiated by the theoretical analysis provided. **Reviewer Summary Questions**

- *Is the work clearly and accurately presented, and does it cite the current literature?*
**Revised to address gaps and balance citations.**
- *Is the study design appropriate, and does the work have academic merit?*
**Clarified as a theoretical and simulation-based study.**
- *Are sufficient details provided for replication?*
**Clarified as numerical reproducibility, not experimental validation.**
- *Are the conclusions supported by the results?*
**Rewritten to match analytical scope.**

We sincerely thank the Reviewer for highlighting critical issues related to reliability engineering, modeling assumptions, and scientific responsibility. The revised manuscript reflects a more mature, careful, and accurate presentation of the work, and we believe it is now suitable for consideration as a theoretical and simulation-based contribution to LiFi research.

# Reviewer report: Reviewer response for version 1

**DOI:** 10.21956/openreseurope.23373.r68130

**Published:** 2026-01-28

**Authors:** MOHAMMAD FURQAN ALI

**Affiliations:** 1 Entomology, University of Agriculture Faisalabad Faculty of Agriculture (Ringgold ID: 467812), Faisalabad, Punjab, Pakistan

**1. Executive Summary**

 The article presents a robust simulation of Direct-Modulated Laser (DML) LiFi systems for clinical settings, demonstrating superior BER ($10^{-79}$) and SNR performance. However, the report identifies a significant real-world gap: the system's reliance on precise optical alignment and complex maintenance makes it vulnerable during "Black Swan" events (natural disasters or conflict). To be scientifically and practically sound for critical infrastructure, the work must address system redundancy and independence from external premises.

**2. Points Must Be Addressed (Required for Soundness)**

**A. Resilience and Disaster Recovery (The "Critique" Point)**

 The authors argue for LiFi as a replacement or primary system. However, in a hospital environment during a disaster (e.g., earthquake or war-time structural damage), laser-based LiFi is highly susceptible to misalignment and power failure.

- **Correction Required:** The authors must discuss or model a **Hybrid LiFi-RF (Radio Frequency) handover**. While LiFi handles high-speed/EMI-sensitive data during normal operations, a simple, ruggedized RF backup (such as Sub-GHz radio) must be integrated to ensure "fail-safe" communication when the optical link is disrupted.

**B. On-Premise Independence**

 The current model assumes a functioning backhaul. If external infrastructure is destroyed, a hospital must function as an "information island."

- **Correction Required:** The authors should specify how the system maintains 100% independence from outer-premise maintenance. This includes the use of Local Edge Computing to process data internally via LiFi even when external telecommunications are severed.

**3. Detailed Assessment**

- **Methods:** The use of OptiSystem and MATLAB is technically sound for establishing the "Best Case" performance metrics of DML-LiFi.
- **Results:** The optimization of launch power ($\ge +5$ dBm) and modulation index ($0.8–1.0$) provides clear engineering guidelines.
- **Conclusions:** The conclusion that DML-LiFi is a "complete" solution is currently overstated. It is a superior *performance* solution but a fragile *resilience* solution unless coupled with legacy radio systems.

**4. Supporting References & Literature (Addressing Resilience)**

 To support the requirement for hybrid systems and independent hospital networks in critical scenarios, the following literature is recommended for inclusion:

**1. On Hybrid LiFi/RF Resilience:**

 This study demonstrates that while LiFi provides speed, RF provides the "coverage and reliability" necessary when the optical path is blocked or the system is under stress.

- **Reference:** Wu, X., et al. (2021). "Dynamic Load Balancing for Hybrid LiFi and Wi-Fi Networks."
- **DOI:** 10.1109/TCOMM.2019.2962434

**2. On Critical Infrastructure Independence:**

 Discusses the necessity of localized "Edge" networks in hospitals to ensure that life-saving data remains accessible even if the external "outer premise" network fails.

- **Reference:** Sahni, Y., et al. (2017). "Edge Mesh: A New Paradigm to Enable Distributed Intelligence in IoT." (Relevant to hospital independent comms).
- **DOI:** 10.1109/ACCESS.2017.2739804

**3. On FSO for Disaster Recovery:**

 Using Free Space Optics (FSO) as a ruggedized, independent backhaul for hospitals when fiber lines are cut during natural disasters.

- **Reference:** Khalighi, M. A., & Uysal, M. (2014). "Survey on Free Space Optical Communication: A Communication Theory Perspective."
- **DOI:** 10.1109/COMST.2014.2329501

**Final Recommendation: **Partly Accept. The article is technically excellent regarding laser physics but requires a "System Resilience" section to be viable for hospital policy implementation. Incorporating a Hybrid RF-Radio backup strategy will make the research significantly more powerful.

Is the study design appropriate and does the work have academic merit?

Yes

Is the work clearly and accurately presented and does it cite the current literature?

Partly

If applicable, is the statistical analysis and its interpretation appropriate?

Yes

Are all the source data underlying the results available to ensure full reproducibility?

Yes

Are the conclusions drawn adequately supported by the results?

Partly

Are sufficient details of methods and analysis provided to allow replication by others?

Yes

Reviewer Expertise:

I am an agricultural entomologist specializing in multidisciplinary research that integrates the principles of physics and chemistry with the life sciences to evaluate system performance in high-stakes environments. My expertise lies in the "Practical Application Validation" of emerging technologies, specifically assessing how complex systems—from biological ecosystems to optical communication networks—function under extreme stress, such as natural disasters or conflict-induced infrastructure failure. My research focuses on building resilient, "fail-safe" architectures that ensure decentralized independence and operational continuity in mission-critical settings, bridging the gap between theoretical laboratory findings and rugged, real-world survival

I confirm that I have read this submission and believe that I have an appropriate level of expertise to confirm that it is of an acceptable scientific standard, however I have significant reservations, as outlined above.

## References

[1] : Edge Mesh: A New Paradigm to Enable Distributed Intelligence in Internet of Things. *IEEE Access* .2017;5: 10.1109/ACCESS.2017.2739804 16441-16458 10.1109/ACCESS.2017.2739804

[2] : Load Balancing for Hybrid LiFi and WiFi Networks: To Tackle User Mobility and Light-Path Blockage. *IEEE Transactions on Communications* .2020;68(3) : 10.1109/TCOMM.2019.2962434 1675-1683 10.1109/TCOMM.2019.2962434

[3] : Survey on Free Space Optical Communication: A Communication Theory Perspective. *IEEE Communications Surveys & Tutorials* .2014;16(4) : 10.1109/COMST.2014.2329501 2231-2258 10.1109/COMST.2014.2329501

# Response

**Published:** 2026-02-09

**Authors:** Ajay Sharma

**Affiliations:** Computer Information Systems, University of Malta Faculty of Information and Communications Technology, Msida, Malta

**Response to Reviewer 1 Comments**

 We are grateful to the reviewer for critically analysing our manuscript and providing positive feedback, which strengthened our work scientifically and practically. The revised manuscript has taken into consideration all the suggestions as explained below.

**Comment 1: Executive Summary** **Reviewer Comment:**

 The manuscript presents strong simulation results but identifies a real-world gap related to system fragility during “Black Swan” events. The work must address redundancy and independence from external premises.

**Response:**

 We concur with the evaluation of the reviewer. We have, in order to close this gap, placed a new dedicated section, namely “System Resilience and Fail-Safe Communication Architecture” **(Section 8).** This part will specifically address system-level resiliency, redundancy, and operational independence, thus making the proposed DML-LiFi system reasonable to support critical infrastructure in hospitals under other than the best-case conditions.

**Comment 2A: Resilience and Disaster Recovery (The “Critique” Point)** **Reviewer Comment:**

 LiFi is susceptible to misalignment and power failure during disasters. A Hybrid LiFi–RF handover must be discussed to ensure fail-safe communication.

**Response:**

**Section 8.1** has discussed this point in detail. The revised manuscript has very clearly placed DML-LiFi as the main high-performance and EMI-safe communication layer, and a low-rate and rugged RF backup to maintain fail-safe operation in case the optical link is broken. The suggested literature on hybrid LiFi/RF supports the discussion **[Wu & Haas]**

**Comment 2B: On-Premise Independence** **Reviewer Comment:**

 The system must maintain independence from external infrastructure so that the hospital can function as an “information island.”

**Response:**

 This has been covered in **Section 8.2**, in which it is explicitly assumed that the system is deployed together with on-premise edge computing resources. This guarantees that critical clinical data processing, storage, and access capabilities are still accessible in the case of unavailability of external telecommunications, as had been previously done on edge-based critical infrastructure networks **[Sahni et al.]**.

**Comment 3: Conclusions** **Reviewer Comment:**

 The conclusion that DML-LiFi is a “complete” solution is overstated unless coupled with legacy radio systems.

**Response:**

 We concur and have amended the Conclusion (it has become Section 9). The absolute statements have been diluted, and the conclusion now clearly states that DML-LiFi is best adopted when embedded into a robust hybrid architecture, including a reliable backup connection that is fail-safe, as explained in Section 8.

**Comment 4: Supporting References & Literature** **Reviewer Comment:**

 Hybrid LiFi/RF systems, critical infrastructure independence, and FSO-based disaster recovery literature should be included.

**Response:**

**Section 8** has included all the recommended references and cited them.

- Hybrid LiFi/RF resilience **[Wu & Haas]**
- Edge-based independent hospital networks **[Sahni et al.]**
- FSO for disaster recovery backhaul **[Khalighi & Uysal]**

The reviewer is once again thanked for the feedback, which has substantially enhanced the relevance and applicability of the manuscript at the system level and for its application to hospital policy and deployment.

# Response

**Published:** 2026-02-23

**Authors:** Ajay Sharma

**Affiliations:** Computer Information Systems, University of Malta Faculty of Information and Communications Technology, Msida, Malta

We are grateful to the reviewer who critically analyzed our manuscript and provided positive feedback, which made our work scientifically and practically sound. The revised manuscript has taken into consideration all the suggestions as explained below. **Comment 1: Executive Summary** **Reviewer Comment:**

 The manuscript presents strong simulation results but identifies a real-world gap related to system fragility during “Black Swan” events. The work must address redundancy and independence from external premises. **Response:**

 We concur with the evaluation of the reviewer. We have, in order to close this gap, placed a new dedicated section, namely “System Resilience and Fail-Safe Communication Architecture” **(Section 8).** This part will specifically address system-level resiliency, redundancy, and operational independence, thus making the proposed DML-LiFi system reasonable to support critical infrastructure in hospitals under other than the best-case conditions. **Comment 2A: Resilience and Disaster Recovery (The “Critique” Point)** **Reviewer Comment:**

 LiFi is susceptible to misalignment and power failure during disasters. A Hybrid LiFi–RF handover must be discussed to ensure fail-safe communication. **Response:**

**Section 8.1** has discussed this point in detail. The revised manuscript has very clearly placed DML-LiFi as the main high-performance and EMI-safe communication layer, and a low-rate and rugged RF backup to maintain fail-safe operation in case the optical link is broken. The suggested literature on hybrid LiFi/RF supports the discussion **[Wu & Haas]** **Comment 2B: On-Premise Independence** **Reviewer Comment:**

 The system must maintain independence from external infrastructure so that the hospital can function as an “information island.” **Response:**

 This has been covered in **Section 8.2**, in which it is explicitly assumed that the system is deployed together with on-premise edge computing resources. This guarantees that critical clinical data processing, storage, and access capabilities are still accessible in the case of unavailability of external telecommunications, as had been previously done on edge-based critical infrastructure networks **[Sahni et al.]**. **Comment 3: Conclusions** **Reviewer Comment:**

 The conclusion that DML-LiFi is a “complete” solution is overstated unless coupled with legacy radio systems. **Response:**

 We concur and have amended the Conclusion (it has become Section 9). The absolute statements have been diluted, and the conclusion now clearly states that DML-LiFi is best adopted when embedded into a robust hybrid architecture, including a reliable backup connection that is fail-safe, as explained in Section 8. **Comment 4: Supporting References & Literature** **Reviewer Comment:**

 Hybrid LiFi/RF systems, critical infrastructure independence, and FSO-based disaster recovery literature should be included. **Response:**

**Section 8** has included all the recommended references and cited them.

- Hybrid LiFi/RF resilience **[Wu & Haas]**
- Edge-based independent hospital networks **[Sahni et al.]**
- FSO for disaster recovery backhaul **[Khalighi & Uysal]**

The reviewer is once again thanked for the feedback, which has substantially enhanced the relevance and applicability of the manuscript at the system level and for its application to hospital policy and deployment.
